# Brain perfusion imaging with voxel-based analysis in secondary progressive multiple sclerosis patients with a moderate to severe stage of disease: a boon for the workforce

**DOI:** 10.1186/s12883-016-0605-4

**Published:** 2016-05-26

**Authors:** Mina Taghizadeh Asl, Reza Nemati, Negar Chabi, Hooman Salimipour, Iraj Nabipour, Majid Assadi

**Affiliations:** Department of Nuclear Medicine, Kasra Hospital, Tehran, Iran; Division of Neuroscience, The Persian Gulf Nuclear Medicine Research Center, Bushehr University of Medical Sciences, Bushehr, Iran; Department of Neurology, Bushehr Medical University Hospital, Bushehr University of Medical Sciences, Bushehr, Iran; Division of Biomedical Engineering (BME), The Persian Gulf Nuclear Medicine Research Center, Bushehr University of Medical Sciences, Bushehr, Iran; The Persian Gulf Tropical Medicine Research Center, Bushehr University of Medical Sciences, Bushehr, 3631 Iran; Department of Molecular Imaging and Radionuclide Therapy (MIRT), Bushehr Medical University Hospital, Bushehr University of Medical Sciences, Bushehr, Iran

**Keywords:** Multiple sclerosis (MS), 99mTc-ECD brain perfusion SPECT, Hyperbaric oxygen therapy (HBOT)

## Abstract

**Background:**

The present study was carried out to evaluate cerebral perfusion in multiple sclerosis (MS) patients with a moderate to severe stage of disease. Some patients underwent hyperbaric oxygen therapy (HBOT) and brain perfusion between before and after that was compared.

**Methods:**

We retrospectively reviewed 25 secondary progressive (SP)-MS patients from the hospital database. Neurological disability evaluated by Expanded Disability Status Scale Score (EDSS). Brain perfusion was performed by (99 m) Tc-labeled bicisate (ECD) brain SPECT and the data were compared using statistical parametric mapping (SPM). In total, 16 patients underwent HBOT. Before HBOT and at the end of 20 sessions of oxygen treatment, 99mTc-ECD brain perfusion single photon emission computed tomography (SPECT) was performed again then the results were evaluated and compared. Brain perfusion was performed by (99 m) Tc-labeled bicisate (ECD) brain SPECT and the data were compared using statistical parametric mapping (SPM).

**Results:**

A total of 25 SP-MS patients, 14 females (56 %) and 11 males (44 %) with a mean age of 38.92 ± 11.28 years included in the study. The mean disease duration was 8.70 ± 5.30 years. Of the 25 patients, 2 (8 %) had a normal SPECT and 23 (92 %) had abnormal brain perfusion SPECT studies. The study showed a significant association between severity of perfusion impairment with disease duration and also with EDSS (*P* <0.05). There was a significant improvement in pre- and post-treatment perfusion scans (*P* <0.05), but this did not demonstrate a significant improvement in the clinical subjective and objective evaluation of patients (*P* >0.05).

**Conclusions:**

This study depicted decreased cerebral perfusion in SP-MS patients with a moderate to severe disability score and its association with clinical parameters. Because of its accessibility, rather low price, practical ease, and being objective quantitative information, brain perfusion SPECT can be complementing to other diagnostic modalities such as MRI and clinical examinations in disease surveillance and monitoring. The literature on this important issue is extremely scarce, and follow up studies are required to assess these preliminary results.

## Background

Multiple sclerosis (MS) is an inflammatory demyelinating disease of the central nervous system (CNS) that affects myelin, oligodendrocytes, and axons, and eventually results in neuronal loss and progressive neurologic disability [1].

In total, four disease courses have been distinguished: relapsing remitting (RR), primary progressive (PP), secondary progressive (SP) and also progressive-relapsing (PR) [2]. The RR type is constituted as the common form of the disease. About two-thirds of patients with RR-MS change in the SP phase where neurologic disability has a progressive pattern between attacks [2, 3].

In the 1930s, Putnam explained the evidence of vascular occlusion in MS histopathology for the first time, and believed that vascular occlusion and inflammation were involved as antecedent processes in demyelinating disease evolution [4]. Wakefield et al. later reported similar findings and showed fibrin deposition and vessel thrombosis without obvious cellular infiltration, which suggested that the presentation of thrombosis in small veins and capillaries supported the possible role of ischemic process in MS disease [5].

Luke et al., who used single photon emission computed tomography (SPECT), showed hypoperfusion of cerebral blood flow (CBF) in the gray matter (GM) of the frontal lobe of patients with progressive MS compared with cerebral perfusion in control patients, although the study reported normal perfusion in patients with RR-MS [6]. Our previous study has similar results and normal brain perfusion of MS patients in the early stage and without significant disability [7]. In another study, Brooks et al. showed that there was decreasing CBF and oxygen utilization in both the white matter and the cortical GM in the MS patients compared to the healthy control group [8].

Some claims exist about the efficacy of HBOT in MS patients. Fisher et al. [9–11], in a double-blind controlled study, showed significant but transient improvements in mobility, fatigability, and walking in MS patients receiving HBOT. Warren et al., showed that HBOT resulted in an augmentation of experimental allergic encephalomyelitis, an animal model of MS [12]. Although different studies had been carried out [13, 14], the assessment of such therapy with functional modalities is scare in the literature. What’s more, recent clinical trial researches along these lines and provided evidence that HBOT can get better the symptoms and life quality of in some neurological disorders [15–25].

We aimed to evaluate cerebral perfusion in MS patients with a moderate to severe stage of disease. Besides, we provided firm appraisal of the HBOT effect on brain activity as assessed by SPECT imaging supplied by quantitative methods and its possible correspondence on clinical status of MS patients.

## Methods

### Participants and study design

We retrospectively reviewed 25 SP-MS patients from the hospital database. The diagnosis was accomplished in agreement with the “McDonald Criteria” [26]. Disability was assessed by a single experienced neurologist blind to the magnetic resonance imaging (MRI) findings using the Expanded Disability Status Scale (EDSS) score [27].

Patients who had one new episode (within 1 month) of acute otitis or those with chronic otitis (three episodes or more within the previous year) were excluded, as were those with any condition that put them at risk of complications from HBOT (e.g. asthma, convulsions). Patients with behavioral problems or those who had had orthopedic surgery (within the prior 6 months), or dorsal rhizotomy within the past 2 years, were also excluded. Previous exposure to HBOT was also an exclusion criterion.

Of these 25 patients, 16 met the criteria and would undergo HBOT. In general, HBOT consisted of 100 % oxygen at a pressure of 1.75 atm absolute (ATA) for 60 min. At the conclusion of 20 sessions of HBOT, brain perfusion SPECT were performed again; the results were evaluated and compared with previous SPECT results.

This study was approved by the ethics committee of the Bushehr University of Medical Sciences.

#### Imaging protocols

About one hour after an IV injection of 740 MBq (20 mCi) 99mTc-ECD (AEOI, Tehran, Iran), the brain SPECT was done. A SPECT equipped with a dual head gamma camera (Siemens e-Cam, Germany), a pair of low-energy and high-resolution collimators was done. Projections were done in a 64 × 64 matrix at 64 steps, 30 s each step. Attenuation correction was done by the Chang manner (attenuation coefficient 0.12 cm^−1^). The projections were then processed using back projection and Butterworth filter (Nyquist frequency cutoff = 0.5, order = 5). Images were presented in three orthogonal planes.

### Image interpretation

#### Visual analysis

Brain SPECT studies were analyzed by two experienced nuclear medicine specialists who were blind to the participants’ data. Discrepancies in final impression were dissolved by consensus. The cortical and subcortical parts were systematically evaluated. The abnormality in radionuclide uptake was classified as follows: no decreased uptake, or normal, mild diminished uptake, and moderate and severe diminished uptake.

No focal hypoperfusion or observable asymmetry in cortical or subcortical regions was considered as normal brain SPECT. Abnormal brain SPECT studies were allocated to heterogeneous rCBF with focal hypoperfusion or visible asymmetry in at least two slice series.

More particularly, the assessment was done separately by two nuclear medicine practitioners who compared the brain SPECT data and classified them as either: 1 = no change, 2 = slight change and 3 = significant change. “No change” was defined as no visual difference in the number or size or severity of perfusion defects; “mild change” defined to a diminish in number or size or severity of perfusion defects; “noteworthy change” to a global perfusion increase in addition to lessening of defect numbers or size or severity.

#### Semi-quantitative analysis using statistical parametric mapping (SPM)

At first, all the images were transformed to NIFTI format using XMedCon software. After that for Normalization step, all the images were spatially normalized to a standard stereotactic space according to the Talairach and Tournoux atlas. Spatial normalization was performed using a SPECT template (provided by SPM8) as a reference image. Then, in smoothing step, all the images were smoothed through using an isotropic Gaussian filter in order to get better the signal to noise ratio.

After preprocessing steps mentioned above, each patient image was compared on a voxel by voxel basis with a pool of control group including 13 cases of non-inflammatory non progressive CNS disorders with normal perfusion SPECT using T-test statistical analysis by selecting two sample t-tests as a design, plus selecting the healthy control group and each patient as the first group scans and second group scan respectively. Relative threshold masking was selected and its threshold was left at the default value 0.8, so the mean intensity was computed from those voxels with intensity higher than 0.8 of the entire scan average. Also, proportional scaling global normalization with its default value (50 ml/dl/min) was used to eliminate the differences between global activities among individuals. It scales each image separately such that its global brain activity will be specified value. Each individual scan was compared against the reference control group which yields a contrast T-map testing for regions with decreased activity (hypoperfusion) compared to the control group. Results were obtained using (*p* <.05) corrected for multiple comparisons (FWE: family wise error correction). Voxels with a P value of less than 0.05 were considered to be significantly different.

Finally, SPM image results then were displayed on a three orthogonal brain which is called glass brain. The regions with the most significant decreased perfusion (CBF) obtained by SPM analysis were in agreement with the regions specified by visual analysis of physician blinded to the SPM results.

### Statistical analysis

Continuous variables are presented as mean ± SD, and categorical variables as the absolute values and percentages. Differences in categorical variables were analyzed using chi-square test. Linear correlations among the variables were assessed using Spearman’s linear correlation coefficient. A p value of <0.05 was considered statistically significant. Statistical analysis was performed using an IBM computer and PASW software, version 18.0 (SPSS, Inc., Chicago, USA).

## Results

A total of 25 SP-MS patients, including 14 females (56 %) and 11 males (44 %) with a mean age of 38.92 ± 11.28 years participated in the study. The mean disease duration was 8.70 ± 5.30 years (Table 1). MRI of the patients demonstrated diffuse hyper intense lesions in the brain and also spine involvement.

**Table 1:** Patients’ characteristics of all included patients

Variable	Total patients (25)
Age (year)	38 (range: 21–58)
Male/Female	11/14
Disease duration (year)	8.70 ± 5.30 (range:1–21)
EDSS (range)	4 (2–8)
Abnormal perfusion (% of all) (mild/moderate/severe)	23 (92) (2/20/1)
Number of patients who underwent O2 therapy (% of all)	16 (64)
O2 therapy session	20

Of the 25 patients, 2 (8 %) have a normal SPECT and 23 (92 %) have abnormal brain perfusion SPECT studies. Brain perfusion SPECT in the abnormal group included: 2 (8 %), 20 (80 %) and 1 (4 %), which showed mild, moderate and severe perfusion scans, respectively (Table 1).

**Fig. 1:**
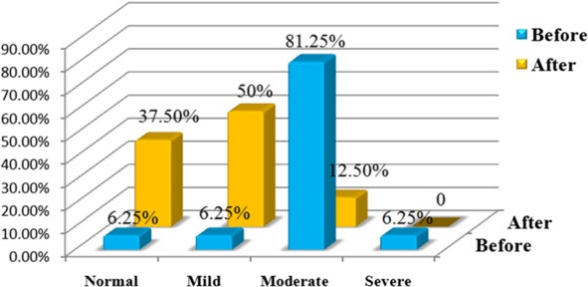
The percent of MS patients based on severity of hypoperfusion in pre-treatment (*left side*) and post-treatment (*right side*) on brain perfusion 99mTc-ECD SPECT images

**Fig. 2:**
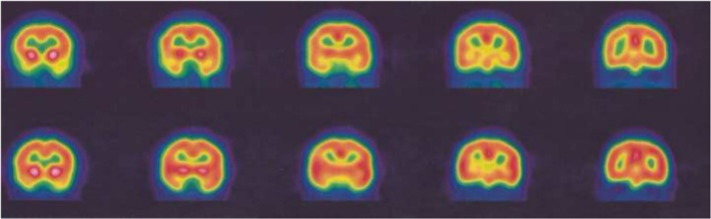
Pre-treatment (*upper rows*) and post-treatment (*lower rows*) SPECT images of a 58-year-old MS patient. There is a moderate hypoperfusion in the posterior right temporal cortex on pretreatment 99mTc-ECD brain perfusion SPECT, which is normalized after hyperbaric oxygen therapy (HBOT). The EDSS of the patient was 6.00 and her disease duration was about 13 years

**Fig. 3:**
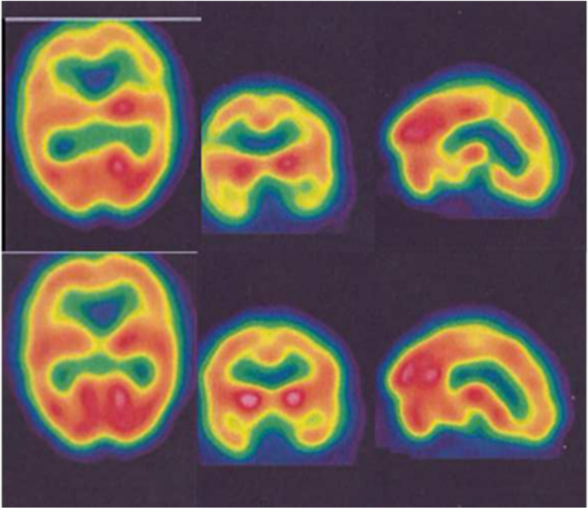
Pre-treatment (*upper rows*) and post-treatment (*lower rows*) SPECT images of a 45-year-old MS patient. There is moderate hypoperfusion in the temporo-parietal regions and caudate nucleus (R>L) on pretreatment 99mTc-ECD brain perfusion SPECT, which is almost completely normalized after hyperbaric oxygen therapy (HBOT). The left columns indicate transverse 99mTc-ECD SPECT images; the middle columns indicate coronal 99mTc-ECD SPECT images; and the right columns indicate sagittal 99mTc-ECD SPECT images. The EDSS of the patient was 6.00 and her disease duration was about 11 years

**Fig. 4:**
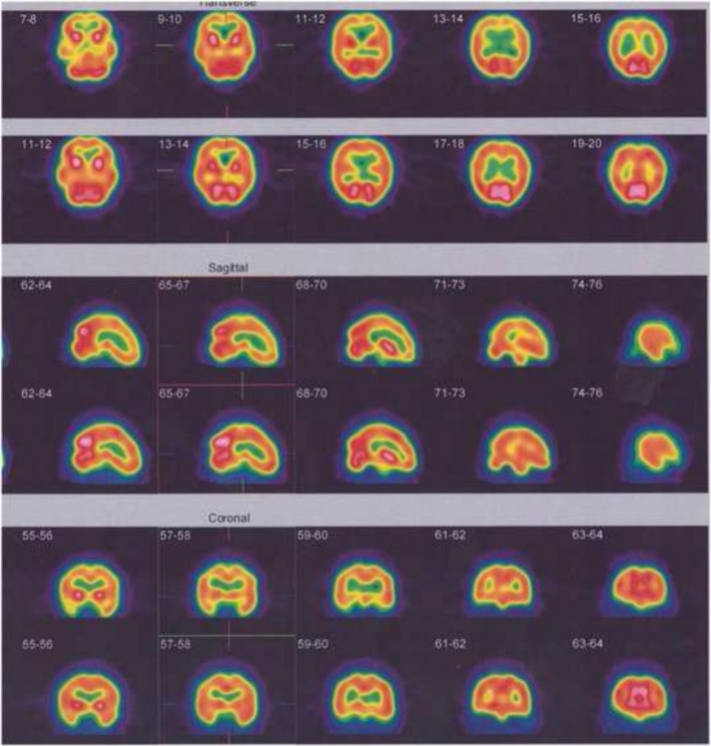
Pre-treatment (*upper rows*) and post-treatment (*lower rows*) SPECT images of a 51-year-old MS patient. There is mild to moderate hypoperfusion through the fronto- parietal cortices bilaterally and sub-cortical structures on pretreatment 99mTc-ECD brain perfusion SPECT, which is almost completely normalized after hyperbaric oxygen therapy (HBOT). The upper rows indicate transverse 99mTc-ECD SPECT images; the middle rows indicate sagittal 99mTc-ECD SPECT images; and the lower rows indicate coronal 99mTc-ECD SPECT images. The EDSS of the patient was 4.00 and her disease duration was about 5 years

In total, 16 patients were treated with HBOT (Table 2). The brain perfusion scans that were obtained before and after therapy showed a significant improvement in perfusion scan results (*P* <0.05) (Figs. 1, 2, 3 and 4). However, the HBOT did not demonstrate a significant improvement in clinical subjective and objective evaluation of the patients (*P* >0.05).

**Table 2:** Characteristics of MS patients who underwent hyperbaric oxygen therapy (HBOT)

Patient no.	EDSS	Interval time (day)	Disease duration (year)	Pre-treatment scan	Post treatment scan
1	4	90	9	Moderate bifrontoparital	Mild bifrontoparital
2	5	30	8	Severe Rt hemisphere	Mild Rt hemisphere
3	3	30	4	Moderate diffuse cortical	Mild diffuse cortical
4	6	50	10	Mild Rt hemisphere	Normal
5	2	240	7	Moderate Rt hemisphere	Moderate Rt hemisphere
6	2	52	2	Normal	Normal
7	3	60	6	Moderate diffuse (L > R)	Moderate diffuse (L > R)
8	3	32	2	Moderate Rt temporal	Moderate Rt temporal
9	3	50	3	Moderate bi temporal	Mild bitemporal
10	6	70	13	Moderate Rt posterior temporal	Normal
11	6	65	11	Moderate bi temporoparital and caudate nucleus	Normal
12	8	26	7	Moderate Rt occipital	Moderate Rt occipital
13	3	60	7	Moderate Rt occipital	Moderate Rt occipital
14	8	60	1	Moderate Lt occipital and putamen	Mild Lt occipital and putamen
15	4	40	5	Moderate bifrontoparital and diffuse subcortical	Normal
16	2	60	5	Moderate Lt occipital	Normal

*EDSS* expanded disability status scale

**Fig. 5:**
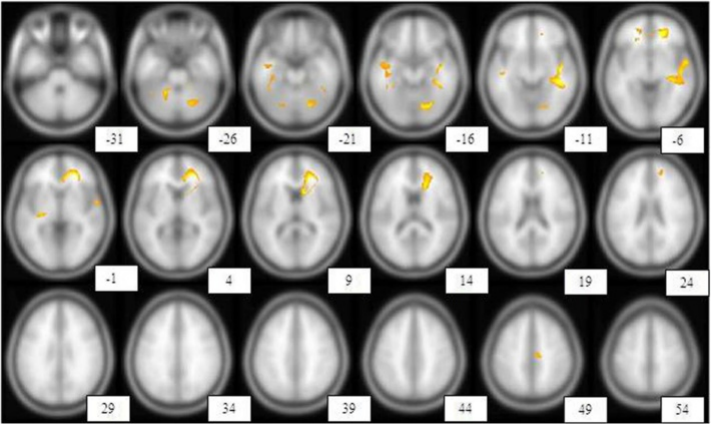
Shows the brain regions with reduced perfusion in one of the MS patients compared with the healthy control group. With a height threshold of *P* <0.05 (corrected for multiple comparison, T = 7.18), there was significant hypoperfusion in different brain areas including right anterior cingulate gyrus, right superior temporal gyrus, left fusiform gyrus, right insula, left inferior frontal gyrus, left anterior cingulate gyrus, left middle temporal gyrus, right supplementary motor area, right lobule IV, V of cerebellar hemisphere, left putamen, left thalamus, left lobule VI of cerebellar hemisphere, right hippocampus, right caudate nucleus. These regions are displayed on transaxial images. Hypoperfusion regions were displayed according to a specified threshold (*p* <0.05, corrected, T = 7.182) image indicates distance (mm) from anterior commisure– posterior commisure plane

Obtained hypoperfusion regions in a patient with multiple sclerosis compared with healthy control group through SPM was mapped on T1 MRI transaxial images (Fig. 5).

The study showed a significant association of severity of perfusion impairment with disease duration and also with EDSS (*P* <0.05).

The time interval between the two before- and after-treatment SPECT examinations was 30–180 days (mean time interval, 64.43 ± 38.66 days).

**Fig. 6:**
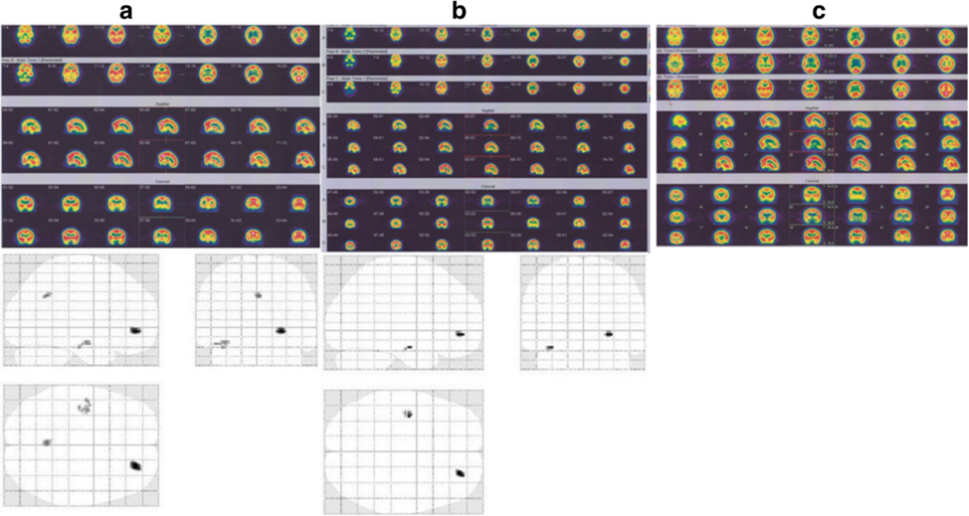
Brain Perfusion SPECT and statistical parametric mapping analysis of a MS patient underwent HBOT. First brain perfusion study of MS woman patient showed moderate hypoperfusion of inferior temporal lobe on the right side (**a**). Second brain perfusion study 33 days after first study and following first oxygen therapy showed mild and moderate hypoperfusion of inferior temporal lobe on the right side at first scan (no significant change in visual analysis) (**b**). Third brain perfusion study 4.4 months after second study and following second oxygen therapy showed rather normal radiotracer distribution through the cortical and subcortical regions (significant changes on visual analysis) (**c**). The upper rows indicate transverse 99mTc-ECD SPECT images; the middle rows indicate sagittal 99mTc-ECD SPECT images; and the lower rows indicate coronal 99mTc-ECD SPECT images

The SPM results table plus its corresponding glass brain of a patient with two subsequent HBOT sessions were presented. There are three follow up results indicating therapy was effective and also based on the obtained figures the regions with significantly reduced perfusion became smaller and smaller and finally in the last scan there was no significant hypoperfused region where the results are in alignment with the visual physician assessment (Tables 3 and 4) (Fig. 6).

**Table 3:** Brain regions with significantly reduced perfusion using statistical parametric mapping analysis, Height threshold, T = 7.26, {*p* <0.05 (FWE)}

*p* (FWE-corr)	T	Z	Coordinates x, y, z {mm} max. peak	Brain area
0.009429	9.117918	4.899239	26, 46, −6	Right superior frontal gyrus, orbital part
0.021354	8.183681	4.672336	−0, −54, 34	Left precuneus
0.02832	7.874513	4.590642	−38, −10, −18	Medial temporal lobe
0.028749	7.858227	4.586238	−46, −8, −20	Left temporal lobe
0.03078	7.784257	4.566107	−40, −18, −24	Left inferior temporal gyrus

**Table 4:** Brain regions with significantly reduced perfusion using statistical parametric mapping analysis, Height threshold, T = 7.26, {*p* <0.05 (FWE)}

*p* (FWE-corr)	T	Z	Coordinates x, y, z {mm} max. peak	Brain area
0.02072	8.219753	4.681639	26, 46, −6	Right superior frontal gyrus, orbital part
0.026836	7.935467	4.607033	−36, −10, −20	Medial temporal lobe

**Fig. 7:**
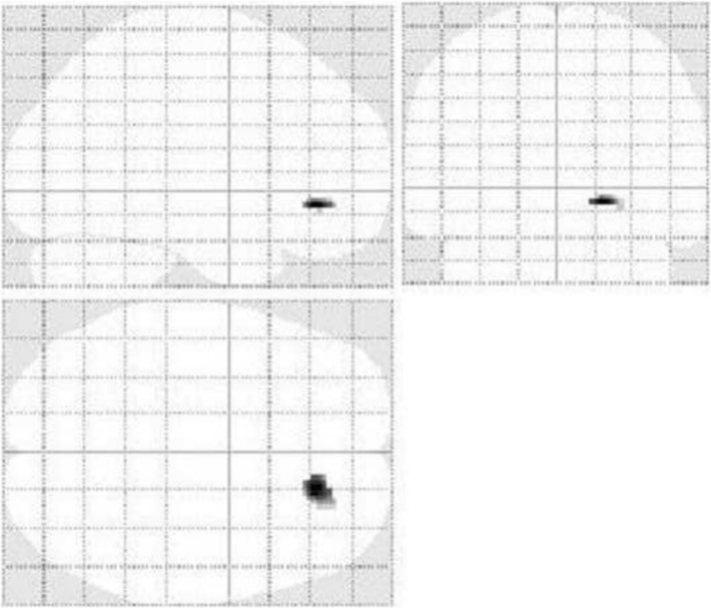
Brain regions with significantly reduced perfusion (statistical parametric mapping) in patients Vs. control subjects (group analysis)

A group semi-quantitative analysis using SPM was performed which clearly showed a significant hypoperfusion in the right medial orbitofrontal cortex (Table 5, Fig. 7).

**Table 5:** Brain regions with significantly reduced perfusion in multiple sclerosis patients (5 patients) compared with control subjects, Height threshold, T = 6.0291, {*p* <0.05 FWE}

*p* (FWEcorr)	T	Z	Coordinates x, y, z {mm} max. peak	Brain area
0.003843	7.936576	4.986516	18, 36, −8	Right medial orbitofrontal cortex

## Discussion

In this study, we investigated cerebral perfusion by SPECT in SP-MS, and evaluated the effects of HBOT in the CBF of these patients. Our study revealed abnormal cerebral perfusion in 92 % of SP-MS patients and for the first time showed that cerebral perfusion significantly improved after HBOT. In addition, it depicted a remarkable association between brain perfusion defects and clinical status. Such findings were also seen on voxel-based analysis of whole-brain data using SPM analysis.

Statistical parametric mapping is a neuroimaging analysis tool which evaluates the statistical significance of cerebral blood flow (CBF) alterations on a voxel by voxel basis, which is more reliable than conventional ROI analysis due to the inherent subjectivity of these methods [28]. SPM was used to identify regions with decreased CBF in each patient compared to the control group [15].

Perfusion studies of MS have different results that may be due to various perfusion measurement techniques and stages of disease [6, 7]. In our previous study, we investigated cerebral perfusion in early RR-MS patients with mild or no disability, which determined normal cerebral perfusion in all patients [7]. Other studies showed different brain perfusion in MS patients particularly in those with a progressive type of disease [6].

By using an arterial spin labeling sequence in early RR-MS patients, Debernard et al. found reduced perfusion in deep GM and different cortical regions, including lingual gyrus, intracalcarine, insular and operculum cortex, temporal, parietal, frontal and occipital cortical regions [29]. In another study, Rashid et al. by arterial spin labeling, found hypoperfusion in several cortical regions of patients with primary and SP-MS but not in patients with RR-MS [30].

Ota et al. assessed the CBF of MS patients by arterial spin labeling and revealed reduced CBF in the bilateral thalami and right frontal region of the patients compared to the healthy volunteers [31]. Lyke et al. evaluated the CBF of MS patients by brain SPECT and showed that CBF was significantly reduced in the patients—particularly in those with the progressive type of MS [6]. Adhya et al. by using dynamic susceptibility contrast perfusion MRI, reported that the CBF was significantly decreased in all normal-appearing white matter (NAWM) regions in the MS patients, and that PP-MS patients showed a significantly lower CBF compared to RR-MS patients [32]. Law et al. determined that the NAWM region of patients with RR-MS had significantly lower CBF compared with the NAWM of control group [4].

Several direct and indirect mechanisms might have contributed to the change in cerebral perfusion of MS patients. The reduction of white matter perfusion may occur following a decrease in tissue metabolism, or it may be due to a primary vascular pathology that may impair cerebral flow [29]. Lower metabolic activity of the cerebral region secondary to neuronal loss (due to the demyelination process) might decrease CBF. Neuronal loss may originate from hypoperfusion, iron deposition, demyelination, or Wallerian degeneration [33]. Neuronal metabolic dysfunction secondary to inflammation with consequent mitochondrial dysfunction may decrease the demand for blood supply and eventually brain perfusion [29]. Axonal transection in the demyelination lesion of the white matter could result in anterograde and retrograde degeneration of axons that are associated with neuronal dysfunction and will consequently decrease GM perfusion [33]. An abnormal cerebral vasculature is another possible mechanism of the GM hypoperfusion [29]. The inflammatory process may cause microvascular damage by several mechanisms. Reaction of cytotoxic T cells with the specific antigen on endothelial surface may start a clotting cascade that eventually results in thrombus formation. Similarly, specific antibodies may react with their antigen on the endothelial cells and result in vascular damage via complement activation. Furthermore, inflammatory vascular edema may interfere with vascular blood flow following focal tissue swelling; whereas acute and chronic venous obliterations may develop as a result of exudation of inflammatory cells and intravascular fibrin deposition [34]. In addition, obliterative vasculitis might cause chronic ischemia resulting from modulation of the vascular tone and the CBF [33, 35]. An increase in the calcium concentration of extracts, neuronal and glial cells shown in MS lesions may be associated with an increase in the tone of blood vessels. Also, a high blood level of endothelin-1, a vasoconstrictor compound, is seen in MS [29, 35]. An abnormal brain cortical-subcortical circuit is considered to be the other mechanism of decreasing brain perfusion in MS patients, which theoretically may be caused by deep GM lesions like the thalamus even by a small lesion that is not obvious [31].

In addition, it revealed a noticeable association between brain perfusion abnormalities and clinical status. In our previous study, we investigated cerebral perfusion in early RR-MS patients with mild or no disability (EDSS score <1) [6]. In contrast, current study depicted significantly cerebral perfusion impairment in MS patients with moderate to severe disability may indicate cerebral perfusion defect increasing with worsening EDSS score. In line with our findings, Lycke et al. [5] evaluated cerebral perfusion in 19 patients with MS and visualized low cerebral perfusion in the frontal grey matter correlated with neurological disability and low perfusion in frontal grey and white matter correlated with impaired cognitive functions. Up to now, clinical and magnetic resonance imaging examinations considered as routine tools to predict disability progression and then appropriate management in MS, however no single parameter is expected to have sound prognostic significance due to complex pathogenesis of MS. What’s more, no conventional MRI procedures has shown strong correlation with long-term disability progression in MS patients, while more developed MRI techniques are currently being investigated with promising results in preclinical settings [35]. The relation between EDSS score and perfusion defect suggest that brain perfusion defect may be helpful parameter to monitor clinical disability and eventually disease progression in MS patient and brain perfusion SPECT can be complementary to other diagnostic modalities such as MRI and clinical examinations in disease surveillance and monitoring. We didn’t evaluated association between MRI lesions and perfusion defect because beyond the scope of our study and should be considered in follow up studies.

Based on the group analysis, right medial orbito frontal cortex was obtained as the significant perfused area among the multiple sclerosis patients, we offered that if the study extended to more patients with MS we may have more precise areas as defected regions. In addition, the aforementioned region was demonstrated on our prior studies that had an important role in status of olfactory. It may amend to do a follow up study in MS patients [33, 36].

Although our study didn’t show a significant clinical benefit from HBOT on the MS patient, the treatment significantly improved the patients’ cerebral perfusion. Another study showed similar results in a clinical setting [34]. In a small, randomized, placebo-controlled trial of HBOT, Martin et al. showed a transient mild improvement in CNS functions, including mobility, coordination, and bladder control, and a reduction in fatigability that is associated with changes of two or more points on the Kurtzke disability status scale [34]. A study by Barnes et al. showed that HBOT in 120 patients with chronic MS was not associated with improvement on the Kurtzke disability status scale [37]. Kindwall et al. didn’t show any benefit from HBOT in MS patients compared to the control group, except for a temporary improvement in bladder control which was reported by some patients [38]. Furthermore, two other main studies did not demonstrate a beneficiary from HBOT in MS patients [9, 38].

The clinical imaging dissociation may originate from the object that old lesions with severe tissue injury in a specific region of the CNS, such as the brainstem and spinal cord, are not affected by HBOT and consequently are not beneficial from the treatment. However, to our knowledge, no functional brain imaging research exists addressing this question in MS subgroups.

The mechanism of the HBOT effect is uncertain. One possibility is an immunosuppressive effect. Another possibility is that ischemic areas of perivenular plaques, which come from an associated venous occlusion, are transiently resolved by HBOT [8]. At the cellular level, HBOT seems to ameliorate neuronal and glia cells’ mitochondrial function and eventually cellular metabolism, reduces oxidative stress, augments neurotrophins and nitric oxide concentrations, and up-regulates axon guidance agents. The treatment may also induce neurogenesis and reduce apoptosis [38]. Another possible mechanism is that hyperoxia acts as a neuronal stimulant [39]. Hyperoxia decreases membrane conductance and affects ion channel closures, presumably decreasing outward (K) and/or inward (Cl) currents that eventually causing depolarization and stimulated to fire rate [39].

All of the mechanisms can promote neuronal activity and metabolism, and eventually increase cerebral perfusion [40–43]. However, there are ongoing studies on HBOT with promising results at least based on imaging findings indicating it will be a open argument in various neurological disorders [15, 16, 44, 45].

In summary, the data may depict diminished cerebral perfusion in SP-MS patients with a moderate to severe disability score and its remarkable association with clinical parameters. Because of its accessibility, rather low price, practical ease, and possibility to acquire objective quantitative information, brain perfusion SPECT can be complementary to other diagnostic modalities in disease surveillance and monitoring, even though additional neurophysiological and imaging investigations are needed. Besides, it might depict the effect of hyperbaric oxygen therapy in the improvement of cerebral perfusion in MS patients but not in clinical outcome. However, it could keep the physiological debate open in which this approach may have some value. The literature on this important issue is scarce, and follow up studies are required to assess these preliminary results.

Finally, it should be mentioned that the current study has a number of limitations. One of the most important limitations is the relatively low number of participants; however, it was quite homogenous in terms of the disease severity of patients. Another limitation of the study is that we didn’t assess the possible association of the cognitive status of MS patients with the improvement in brain perfusion following HBOT mostly due to retrospective nature of this study. Furthermore, most brain perfusion studies showed improvement following HBOT without a substantial effect on clinical subjective and objective evaluation in the short-term follow up. Extended monitoring in a more prolonged time course should be taken into account in future studies.

## Conclusion

This study demonstrated decreased cerebral perfusion in SP-MS subjects with a moderate to severe disability score and its association with clinical parameters. Because of its accessibility, rather low price, practical ease, and being objective quantitative data, brain perfusion SPECT could be complementing to other diagnostic modalities such as MRI and clinical examinations in disease surveillance and monitoring, even though additional neurophysiological and imaging investigations are highly required.

## Abbreviations

CNS, central nervous system; EDSS, expanded disability status scale score;HBOT, hyperbaric oxygen therapy; MS, multiple sclerosis; PP, primary progressive; PR, progressive-relapsing; RR, relapsing remitting; SP, secondary progressive; SPM, statistical parametric mapping; Tc-99 m-ECD SPECT, Tc-99 m ethyl cysteinate dimer single-photon emission computed tomography.
